# The Prevalence of Depression and Anxiety in Post-bariatric Surgery Patients at King Khalid University Hospital, Riyadh

**DOI:** 10.7759/cureus.32500

**Published:** 2022-12-14

**Authors:** Sulaiman A Alshammari, Mohammed A Alassiri, Hussain A Allami, Hisham M Almousa, Abdulaziz S Alobaid, Dawood H Ismail, Abdulhakim I Bin Onayq

**Affiliations:** 1 Family and Community Medicine, King Saud University Medical City, Riyadh, SAU

**Keywords:** obesity, saudi arabia, anxiety, depression, bariatric surgery

## Abstract

Background

Obesity negatively impacts mental and physical health and is a leading cause of disease worldwide. Obesity affects 33% of Saudi adults, with 10% being morbidly obese (body mass index, BMI >40 kg/m^2^). This study explored the association between bariatric surgery (BS) and a predisposition or exacerbation of depressive and anxiety symptoms.

Material and methods

A cross-sectional study of patients who underwent bariatric surgery at the King Khalid University Hospital in Riyadh, Saudi Arabia, was conducted between February 2016 and December 2021. The patients were contacted by phone to complete a self-administered questionnaire on demographic information, chronic medical diseases, psychiatric diseases, body mass index, and type of bariatric surgery. In addition, they completed the patient health questionnaire-9 (PHQ-9) and general anxiety disorder-7 (GAD-7) questionnaire to screen for patients’ depression and anxiety symptoms.

Results

The findings of the 367 BS patients showed that 20.7% of the patients were considered to have mild anxiety, 11.2% had moderate anxiety, and 8.7% had high anxiety levels. However, regarding depression, 46.9% had extremely low levels of depression, followed by mild depression in 29.4% and moderate depression in 11.2%. Furthermore, another 8.2% of BS patients had moderately high depression levels, and 4.4% had severe depression. The anxiety and depression levels of the patients in this study did not show any statistically significant changes postoperatively in the short, medium, or long term. On the other hand, almost all of the patients 97% who underwent bariatric surgery were satisfied with the outcome of their surgery.

Conclusion

Few BS patients had high symptoms of depression and anxiety. We recommend pre- and postoperative psychiatric assessment for all bariatric surgery patients as surgical protocol.

## Introduction

Obesity is one of the most prevalent medical problems worldwide, with numerous physical and mental adverse health effects due to its role in the spread of multiple diseases [1-3]. Obesity is defined by the World Health Organization as a body mass index (BMI) of 30 kg/m^2^ or higher [4]. Over 1.9 billion adults who are 18 years old and older were overweight in 2016; of these adults, over 650 million were obese, and there approximately 13% of adults worldwide were obese (11% men and 15% women) between 1975 and 2016, the prevalence of obesity nearly tripled globally [4].

In the US, adult obesity increased from 33.7% in 2007-2008 to 39.6% in 2015-2016 [5]. The prevalence in Saudi Arabia is estimated to be around 33% obese adults, about 10% morbid obesity (BMI >40 kg/m^2^) [6], and recent studies predicted that by 2022 it might reach 59% [6]. Obesity is associated with chronic weight-related somatic comorbidities, such as type 2 diabetes, metabolic syndrome, cardiovascular diseases, obstructive sleep apnea, osteoarthritis, gastroesophageal reflux disease (GERD), hepatobiliary diseases, and polycystic ovary syndrome (PCOS) [7,8]. Due to that, individuals with obesity exhibit impairment in health-related quality of life and a reduction in life expectancy [7,8]. Several studies show that obesity is a high risk of psychological distress, depression and anxiety, and impaired quality of life [9]. Bariatric surgery is effective in controlling weight, lowering overall mortality [10,11], improving weight-related somatic comorbidities, increasing physical health-related quality of life, and long-term survival [12-15], it has also been shown to improve several psychological symptoms [11,16] as well as general health, physical function, social function, vitality, and emotional role [17]. Bariatric surgery is recommended for individuals with class 3 obesity (BMI ≥40 kg/m^2^) or patients with class 2 obesity (BMI ≥35 kg/m^2^) and weight-related somatic comorbidity [18]. Today, Roux-en-Y gastric bypass (RYGB), sleeve gastrectomy (SG), and adjustable gastric banding are the most popular and commonly performed bariatric surgeries (BS) [19]. It is well known that many bariatric surgery candidates suffer from mental health disorders, particularly depression and binge eating disorders [20]. However, the outcomes of bariatric surgery are variable in some studies. They show that depressive symptoms may worsen in some patients [21]. Others suggested that some patients may have a higher chance of depression, anxiety, and other psychiatric illnesses [9]. Further studies have also reported that up to 65% of bariatric surgery patients endorsed a history of depression or mood disturbance [22,23]. Studies focusing on psychological changes and psychiatric disorders among bariatric patients are limited in Saudi Arabia [20,24,25]. Weight loss after bariatric surgery is related to short- and medium-term decreases in depression [9,17,26]. While the latter outcome is encouraging, long-term follow-up data suggest that some postoperative patients do not experience psychological benefits or report de novo development of depression or the return of depressive symptoms that have initially improved after surgery [27]. Elevated levels of depression post-surgery may contribute to the experience of suboptimal outcomes after surgery, including unsatisfactory weight loss or weight regain, comorbid psychopathology, reduced health-related quality of life [28,29], an increased risk for suicidal ideation, suicide attempts, and completed suicides following bariatric surgery [30-33]. This study explored the association between bariatric surgery and an exacerbation or predisposition to depressive and anxiety symptoms.

## Materials and methods

A cross-sectional study of patients who underwent bariatric surgery at the King Khalid University Hospital in Riyadh, Saudi Arabia, was conducted between February 2016 and December 2021. The 367 patients who participated in the study were contacted by phone and informed about the study's goals to get their consent to participate.

After reviewing the literature, we made an electronic, self-administered questionnaire in two parts. The first part assesses demographic information-age, nationality, gender, marital status, education, household income, and place of residence. Furthermore, socioeconomic status, including major events that negatively affect psychological health, such as losing a job or income, deteriorating economic situation, getting divorced, retiring, or experiencing grief or loss, and open questions for any other cause were investigated. Another inquiry was to assess chronic medical diseases such as DM, HTN, dyslipidemia, etc.; family history of psychiatric illness; body mass index; and type of bariatric surgery. The second part consists of two questionnaires to assess patients' anxiety and depression disorders. Depressive symptoms were evaluated using the patient health questionnaire-9 (PHQ-9), which has been extensively validated in many patient populations [34,35]. The PHQ-9 consists of nine items scored from 0 to 3, and the PHQ-9 thresholds for mild, moderate, and severe depression are 5, 15, and 20, respectively.

Similarly, anxiety symptoms were assessed using the general anxiety disorder-7 (GAD-7), which had previously been validated in the general population. In addition, its psychometric properties have been studied in bariatric surgery patient populations [36]. In this seven-item questionnaire, mild anxiety has a score of 5 or less, moderate anxiety has a score between 5 and 10, and 15 or above is considered severe anxiety. In addition, there are some yes-or-no questions, such as did you experience difficulty with the activities of daily living (ADL) due to depression symptoms? Are there any close family member/s who have been diagnosed with psychological/mental illness? And have you experienced family, social, work, or related economic problems recently?

Statistical analysis

The mean and standard deviation were used to describe the continuously measured variables, and the median and interquartile ranges for the continuous variables with skewness. The frequency and percentages were used to describe the categorically measured variables. The Cronbach's alpha test was used to assess the internal consistency of the measured questionnaires 0.91 for GAD-7 and 0.90 for PHQ-9. The bivariate Pearson's correlations test (r) was used to determine the correlations between metric variables. The chi-squared test of independence was used to assess the association between categorically measured variables, with a likelihood-ratio adjusted chi-squared test used for contingency tables that violated the statistical assumption for the chi-squared test. A socioeconomic index was computed with the categorical factor analysis of the patients (educational, household income, occupation, marital status), yielding a standardized socioeconomic factor, or index, with a mean value=0 and SD=1. People with higher scores had greater educational, occupational, economic, and social states and vice versa. The multivariate generalized linear models with gamma regression were applied to the patients' mean perceived depression and anxiety scores. The associations between the patients' measured risk factors and characteristics with those outcomes were expressed as multivariate-adjusted risk rates (RRs) with associated 95% confidence intervals. The SPSS IBM v21 (SPSS, Version 21, IBM, Chicago, USA) statistical data analysis program was used for the data analysis, and the alpha significance level was considered at 0.05.

## Results

Table 1 shows the 367 bariatric surgery patients who completed the online survey. Women made up 58% of the participants. The age distribution of the patients was as follows: 29.2% were 30, 34.9% were 30-39, 19.3% were 40-49, and 16.6% were over 50. Also, 32.7% were never married, but 67.3% were married. Furthermore, 31.6% had a high school or less, while 68.4% were educated beyond high school. In addition, 40.3% of respondents were housewives, unemployed, or students; 22.9% worked in the private sector or were self-employed, and 36.8% worked for the government. The household monthly income (HHMI) for these patients was as follows: 25.3% had HHMI less than 5000 SAR (Saudi Riyals), 27% had HHMI between 5000 and 10,000 SAR, 31.6% had HHMI between 10,000 and 20,000 SAR, and the remainder had HHMI greater than 20,000 SAR. In addition, 76% of patients had never smoked, 6.3% had quit, and 17.7% were current smokers.

**Table 1:** Descriptive analysis of the bariatric surgery patients' sociodemographic characteristics, N=367. Ph.D.: Doctor of Philosophy, SAR: Saudi Riyal.

Sociodemographic characteristics	Frequency (%)
Sex	
Female	213 (58%)
Male	154 (42%)
Age group	
<30 Years	107 (29.2%)
30—39 Years	128 (34.9%)
40—49 Years	71 (19.3%)
≥50 Years	61 (16.6%)
Marital state	
Never married	120 (32.7%)
Ever married	247 (67.3%)
Educational level	
High school or less education	116 (31.6%)
Diploma degree	49 (13٫4%)
University degree	170 (46.3%)
Master's and Ph.D. degree	32 (8.7%)
Occupation/employment status	
Unemployed/housewife	148 (40.3%)
Private sector employed	84 (22.9%)
Governmental sector employed	135 (36.8%)
Household's monthly income in Saudi Riyals	
<5000 SAR	93 (25.3%)
5000—10,000 SAR	99 (27%)
10,000—20,000 SAR	116 (31.6%)
>20,000 SAR	59 (16.1%)
Smoking habit	
Never smoker	279 (76%)
Former smoker	23 (6.3%)
Yes-active smoker	65 (17.7%)

According to Table 2, 38.7% of people had at least one comorbidity, 6.3% had cardiovascular disease, 29.6% had diabetes mellitus, 10.6% had hypothyroidism, 33.8% had hypertension, 23.9% had the respiratory disease (including allergic diseases with asthma), and 21.8% had dyslipidemia. Before BS, 37.9% of patients had sleep apnea, and 89.9% improved after surgery. Concerning snoring, 46.6% of the patients had a positive history of snoring, but 87.7% had improved with post-BS. Furthermore, 64.9% of BS patients had joint pain, but 74.8% improved post-BS. The findings revealed that 56.9% of the patients' esophageal regurgitation and 27.2% improved after the bariatric surgery. However, 20.7% of patients experienced worse symptoms following bariatric surgery, and before the surgery, 33.8% of women had ovarian cysts, but 55.6% had improved post-BS.

**Table 2:** Descriptive analysis of the bariatric surgical patients' comorbidity, sleep apnea, snoring, joint-related pains and problems, esophageal regurgitation, and ovarian cysts, N=367. CKD: chronic kidney disease, SLE: systemic lupus erythematosus.

Variables	Frequency (%)
Comorbidity	
No	225 (61.3%)
Yes	142 (38.7%)
Type of comorbidity	142 (38.7%)
Cardiovascular disease	9 (6.3%)
Diabetes mellitus	42 (29.6%)
Hypothyroidism	15 (10.6%)
Hypertension	48 (33.8%)
Asthma and respiratory disease	34 (23.9%)
Dyslipidemia/high cholesterol	31 (21.8%)
Rheumatoid disease	13 (9.2%)
Other (CKD, joint disease, SLE, psoriasis, disc dislocation, urticaria, and vitamin deficiency)	21 (14.8%)
History of obesity-related health problems	
Sleep apnea	139 (37.9%)
Yes, but had improved and disappeared post-surgically	125 (89.9%)
Yes, but unchanged compared to the pre-operative time	13 (9.4%)
Yes, worsened	1 (0.7%)
Snoring	171 (46.6)
Yes, but had improved and disappeared post-surgically	150 (87.7%)
Yes, but unchanged compared to the pre-operative time	20 (11.7%)
Yes, worsened	1 (0.5%)
Joint-related pains and problems	238 (64.9%)
Yes, but had Improved and disappeared post-surgically	178 (74.8%)
Yes, but unchanged compared to the pre-operative time	31 (13.0%)
Yes, worsened	29 (12.2%)
Esophageal regurgitation	209 (56.9%)
Yes, but had improved and disappeared post-surgically	100 (27.2%)
Yes, but unchanged compared to the pre-operative time	33 (9%)
Yes, worsened	76 (20.7%)
Ovarian cysts	Females only 72 out of 213 (33.8%)
Yes, but had Improved and disappeared post-surgically	40 (55.6%)
Yes, but unchanged compared to the pre-operative time	26 (36.1%)
Yes, worsened	6 (8.3%)

According to Table 3, before the bariatric surgery, 45.2% of patients were obese >40, 30.2% were 35-40, and 24.5% were 30-35. Following surgery, 16.1% had a BMI of <20, 36.2% had a BMI of 20-25%, and 23.7% had a BMI of 25-30%, but 14.4% had a BMI of 30-35%, 6.5% had a BMI of 35-40%, and 3% had a BMI of >40%. BMI was reduced in approximately 94% of post-bariatric surgery patients. The findings revealed that 5.7% had undergone gastric bypass surgery, and the vast majority (94.3%) had undergone gastric sleeve reduction surgery. Regarding the date of their bariatric procedures/surgery, the analysis findings indicated that 9.5% of them had their bariatric procedures within six months of the date of the survey, and another 17.7% had been operated on between six months and one year ago, but 19.1% had been operated on between one to two years ago and 23.7% between two to three years ago. However, 16.3% of the patients had their bariatric procedures within the previous three to four years, and the remaining 13.6% had their bariatric procedures more than four years before the survey time. Furthermore, only 10.4% of the patients had experienced BS-related complications, and when asked to rate their level of satisfaction with the bariatric surgery, the majority, 91.3%, were satisfied with the results.

**Table 3:** Descriptive analysis of the bariatric surgical patients' body mass index, type of bariatric surgery, time of the bariatric surgery, complications, and satisfaction, N=367. BMI: body mass index.

Variables	Frequency (%)
Body mass index level prior to bariatric surgery	
30—35%	90 (24.5%)
35—40%	111 (30.2%)
>40%	166 (45.2%)
Body mass index level post to bariatric surgery	
<20%	59 (16.1%)
20—25%	133 (36.2%)
25—30%	87 (23.7%)
30—35%	53 (14.4%)
35—40%	24 (6.5%)
>40%	11 (3%)
BMI improvement post-bariatric surgery	
Improved	345 (94%)
Not improved	22 (6%)
Type of bariatric surgery received	
Gastric bypass surgery	21 (5.7%)
Gastric sleeve reduction surgery	346 (94.3%)
Time of the bariatric surgery	
<6 Months	35 (9.5%)
6 M—1 year	65 (17.7%)
1—2 Years	70 (19.1%)
2—3 Years	87 (23.7%)
3—4 Years	60 (16.3%)
>4 Years	50 (13.6%)
Experienced any bariatric surgical-related problems/complications	
No	329 (89.6%)
Yes	38 (10.4%)
How satisfied are you with the bariatric surgery outcome, mean (SD)=4.53 (0.82)	
Very dissatisfied	6 (1.6%)
Dissatisfied	8 (2.2%)
Neutral	18 (4.9%)
Somehow satisfied	88 (24.0%)
Very satisfied	247 (67.3%)

According to Table 4, before surgery, 70 (19.1%) of bariatric patients had seen a psychiatrist within the previous four years. Only 19.1% of patients were referred to psychiatrist clinics, and only 9% of the patients were diagnosed with psychiatric illnesses. However, 2.9% of the patients had obsessive disorders, 8.6% had schizophrenia, 17.1% had panic disorders and social phobias, 11.4% had personality and other less common mental disorders, and 57.1% had mild to moderate depression. In addition, 12.8% of patients had a family history of psychiatric/mental illness. According to the analysis, these family members had 11.1% schizophrenia, 8.9% panic attacks, 6.7% obsessive disorders, 2.2% hyperactivity disorders, 62.2% depression, and 28.9% anxiety disorders, and 4.6% of participants reported using marijuana, amphetamines, and other drugs. About 33.8% had social/economic/work/marital problems.

**Table 4:** Descriptive analysis of the bariatric surgical patients' psychological and social conditions, N=367.

Variables	Frequency (%)
Have you visited the psychiatrist clinic prior to surgery	
No	297 (80.9%)
Yes	70 (19.1%)
When did you visit the psychiatrist clinic, n=70	
Visited but can't remember when	37 (52.9%)
Yes, in less than one year	16 (22.9%)
Yes, one to three years before	15 (21.4%)
Yes, four years before surgery	2 (2.8%)
Were you diagnosed with any psychological illness	
No	334 (91.0%)
Yes	33 (9.0%)
What psychological diagnoses did you have	
Obsessive disorder	1 (2.9%)
Schizophrenia	3 (8.6%)
Panic attacks and social phobia	6 (17.1%)
Personality disorders	4 (11.4%)
Other psychiatric illness	4 (11.4%)
Anxiety disorder	9 (25.7%)
Depression	20 (57.1%)
Is there any close family member/s who had been diagnosed with psychological/mental illness?	.
No	320 (87.2%)
Yes	47 (12.8%)
What psychological diagnoses do they have, n=45	
Schizophrenia	5 (11.1%)
Panic attacks	4 (8.9%)
Obsessive-compulsive disorder	3 (6.7%)
Hyperactivity	1 (2.2%)
Depression	28 (62.2%)
Anxiety disorder	13 (28.9%)
Have you used amphetamines and illicit drugs before	
No	350 (95.4%)
Yes	17 (4.6%)
Have you experienced family, social, work, or economic related problems recently	
No	243 (66.2%)
Yes	124 (33.8%)

Table 5 displays the descriptive analysis findings for the patient's overall perceptions of anxiety and depression and their categorized levels based on their scoring methods. The BS patients' mean anxiety score was 5.05 (SD 5.34), a median of 3 points, and an IQR of 7 points, indicating a low level of anxiety. According to the GAD-7 questionnaire scoring system, most BS patients had low/little anxiety, 20.7% had mild anxiety, 11.2% had moderate anxiety, and 8.7% had high anxiety. The BS patients' overall mean depression (PHQ-9) score was 6.43 (SD 6.18), and the median value was 5 (IQR=7). According to the PHQ-9 questionnaire scoring system, 46.9% of patients had very low depression, 29.4% had mild depression, and 11.2% had moderate depression. Conversely, 8.2% of bariatric surgery patients had moderately high depression, and 4.4% had severe depression.

**Table 5:** Descriptive analysis of the patient's overall perceptions of generalized anxiety and depression. GAD-7: general anxiety disorder-7, PHQ-9: patient health questionnaire-9, ADL: activities of daily living.

Variables	Mean	SD	Median	IQR
Generalized anxiety (GAD-7) score	5.05	5.34	3	7
Generalized anxiety level				
Low/little anxiety, n (%)	218 (59.4%)			
Mild anxiety, n (%)	76 (20.7%)			
Moderate anxiety, n (%)	41 (11.2%)			
High anxiety, n (%)	32 (8.7%)			
Patients' health (PHQ-9) score	6.43	6.18	5	7
Depression PHQ-9 level				
Very low depression, n (%)	172 (46.9)			
Mild depression, n (%)	108 (29.4)			
Moderate depression, n (%)	41 (11.2)			
Moderate-high depression, n (%)	30 (8.2)			
Severe depression, n (%)	16 (4.4)			
Perceived difficulty with ADL due to depression and anxiety symptoms	1.4	0.68		

The multivariate analysis showed that the patients' sex had correlated significantly with their mean perceived depression score (Table 6). Male bariatric patients were significantly less predicted to have depression (23.4% times less predicted) compared to females on average, with a p-value <0.001, accounting for the other independent predictor variables in the analysis model. And the multivariate findings showed that the patients' age group had converged significantly but negatively on their measured depression (PHQ-9) mean score, patients aged between 30 and 39 years were found to be significantly less predicted of depression (18.3% times less predicted) compared to those aged <30 years, p-value=0.021, also the patients aged between 40 and 49 years were found to be significantly less predicted to depression (23.8% times less predicted) on average compared to bariatric patients aged <30 years, p-value=0.012, as well the patients aged ≥50 years were found to be significantly less predicted for post-bariatric surgery depression (33% times less predicted) compared to patients aged <30 years on average, p-value <0.001. The patients' bariatric type of surgery did not converge significantly on their mean depression score, p-value=0.945. But the multivariate-adjusted findings showed that the patients who experienced family, work, or economic troubles had experienced significantly higher mean depression PHQ-9 score (1.271 times higher or 27.1% times more depression) on average compared to those bariatric surgery patients who had no such troubles, p-value=0.002. Not only that, but also the patients' mean perceived ADL difficulties due to anxiety symptoms had correlated positively and significantly with their mean depression PHQ-9 score, as the patients' self-rated mean ADL difficulties due to anxiety tended to rise by one point on the Likert-like scale, their mean predicted depression rate tended to increase by a factor equal to 1.379 times higher (or 37.9% times higher) on average, p-value <0.001, and the patients mean perceived ADL difficulties due to depression symptoms had correlated positively and significantly too with their mean depression PHQ-9 score, as the patients self-rated mean ADL difficulties due to depression symptoms tended to increase by one point on the Likert-like scale their mean predicted depression rate tended to rise by a factor equal to 1.326 times higher (or 32.6% times higher) on average, p-value <0.001. The patients who experienced ADL difficulties due to anxiety and depression symptoms predicted significantly higher depression scores for those patients after their BS. The patients' history of sleep apnea did not correlate significantly with their post-bariatric surgery depression score. Still, patients with a positive history of regurgitation and esophageal reflux experienced significantly higher mean depression scores (1.179 times higher, 11.8% times higher) on average compared to patients with no history of regurgitation, p-value=0.026. Also, post-bariatric surgery patients with a positive history of childhood language and speech disorders were significantly more predicted (1.417 times higher) to have depression than those with a negative history of childhood language and speech disorders, p-value=0.006. The patients' mean self-rated satisfaction level with post-bariatric surgery outcomes had correlated slightly negatively, though not statistically significant, with their depression score, p-value=0.054, but their history of illicit drug use did not correlate significantly with their mean depression PHQ-9 score, p-value=0.108. The other measured predictor-independent variables were not statistically significantly correlated with the patient's depression score.

**Table 6:** Multivariate generalized linear model with gamma regression of the bariatric surgery patients' perceived depression (PHQ-9) score and anxiety (GAD-7) score. PHQ-9: patient health questionnaire-9, ADL: activities of daily living, GAD-7: general anxiety disorder-7, SES: socioeconomic factor, BMI: body mass index.

Parameter	Multivariate-adjusted risk rate (RR)	95% Wald CI for (RR)		
		Lower	Upper	p-value
Depression (PHQ-9):				
Intercept	4.018	2.332	6.925	<0.001
Sex, male	0.766	0.664	0.883	<0.001
Age, group ≥50 years	0.670	0.539	0.832	<0.001
Age, group=40—49 years	0.762	0.616	0.943	0.012
Age, group=30—39 years	0.817	0.689	0.969	0.021
Type of bariatric surgery=gastric sleeve reduction surgery	1.011	0.747	1.368	0.945
Underwent recent family/work/economic troubles	1.271	1.092	1.480	0.002
Perceived ADL difficulty from anxiety symptoms score	1.379	1.179	1.612	<0.001
Perceived ADL difficulty from depression symptoms score	1.326	1.133	1.552	<0.001
History of sleep apnea before surgery	1.127	0.971	1.308	0.116
History of regurgitation before surgery	1.179	1.020	1.363	0.026
Self-rated satisfaction with the bariatric surgery outcome score	0.916	0.837	1.001	0.054
Use of illicit drugs	1.305	0.944	1.805	0.108
Anxiety (GAD-7) score				
Intercept	2.472	1.367	4.470	0.003
Sex, male	0.741	0.632	0.867	<0.001
Age, group ≥50 years	0.789	0.623	0.998	0.048
Age, group=40—49 years	0.909	0.718	1.152	0.429
Age, group=30—39 years	0.944	0.785	1.137	0.545
Employed patient (any job)	1.235	1.001	1.524	0.049
Type of bariatric surgery=gastric sleeve reduction surgery	0.822	0.603	1.121	0.215
Perceived ADL difficulty from anxiety symptoms score	1.779	1.512	2.093	<0.001
Perceived ADL difficulty from depression symptoms score	1.276	1.091	1.493	0.002
History of sleep apnea before bariatric surgery	1.191	1.020	1.392	0.027
Socioeconomic factor (SES) score	0.857	0.772	0.952	0.004
Experienced no reduction in BMI post-bariatric	0.628	0.424	0.932	0.021
Self-rated satisfaction with the bariatric surgery outcome score	0.944	0.860	1.037	0.230
Dependent variable: patients perceived generalized anxiety (GAD-7) score. Patient health (PHQ-9) score. Estimation method=Maximum likelihood estimate with gamma regression.				

The multivariate generalized linear models showed that the patients' sex had correlated significantly but negatively with their mean perceived anxiety GAD-7 score. Male patients experienced significantly lower mean anxiety scores (25.9% times less) compared to female patients after their BS on average, p-value <0.001. The patients' age had also correlated significantly with their mean perceived anxiety GAD-7 score, patients aged ≥50 years were found to be significantly less predicted to have anxiety (21.1% times less) on average compared to patients aged <30 years, p-value=0.049, but the patients aged 40-49 years and 30-39 years may not necessarily differ significantly with their mean perceived anxiety when compared to patients aged <30 years on average, p-value >0.050 each, respectively. Nevertheless, on average, the employed patients measured significantly higher mean anxiety post their bariatric surgery (1.235, or 23.5% times higher) than unemployed/housewives, p-value=0.049. The patients' type of received bariatric surgery did not correlate significantly with their perceived anxiety (GAD) score, p-value=0.215. Still, their mean perceived ADL difficulties due to anxiety had correlated significantly and positively with their mean perceived GAD-7 anxiety score, p-value <0.001, also their mean perceived ADL difficulty score due to depressive symptoms had correlated significantly and positively with their mean perceived anxiety GAD-7 score, p-value=0.002, accounting for the other predictor independent variables in the analysis model.

Interestingly, the post-bariatric surgery patients with a positive history of sleep apnea were found to be significantly more inclined to anxiety (19.1% times more) compared to patients with no sleep apnea history before and after the surgery, p-value=0.027. As well, the patients socioeconomic (SES) factor score correlated significantly but negatively with their mean perceived anxiety GAD score; as the patients' measured socioeconomic index score tended to rise by one standard point on average, their mean predicted generalized anxiety GAD-7 score tended to decline significantly by a factor equal to 14.3% times less on average too, p-value=0.004, higher socioeconomic state for those post-bariatric surgical patients predicted significantly lower anxiety in general and by accounting for the other predictor variables in the analysis (Appendix 1, Table 7). The patients who had experienced no improvement in their body mass index (BMI) post-bariatric surgery were found to be significantly less predicted to have anxiety (37.2% times less) compared to those who had indeed lost some body mass after receiving their bariatric surgery, p-value=0.021. However, an interaction effect between the predictors (date of the surgery × no improvised BMI post-surgically) was tested for statistically significant impact on the patients' mean perceived GAD-7 score within the above model, and it showed no statistically significant interaction effect. The mean GAD score is depicted on the y-axis, and the date of bariatric surgery on the x-axis with subgroup analysis in the bars for improvement in the patient's BMI index, it is clear that the patients who did not lose weight post-bariatric surgery had generally measured more anxiety score compared to those who did indeed lose body mass for all levels of surgical date except those operated within the past six months ago, one to two years, and four years ago; those who did not lose BMI within those periods in particular (six months, two years, and four years ago) measured slightly lower mean anxiety compared to those who operated within the same periods. The interaction term was removed from the model due to the lack of a statistically significant effect, and the lack of BMI improvement was kept as a general term. However, the patient's satisfaction with their post-bariatric surgical outcomes did not correlate significantly with their mean perceived GAD-7 anxiety score. The other measured independent predictor variables were not statistically significantly correlated with the patient's anxiety score.

## Discussion

In our study, the majority of BS patients had low or little anxiety levels, 20.7% of the patients were considered to have mild anxiety, 11.2% of them were regarded as moderate anxiety, and 8.7% were considered to have high anxiety levels. In the survey, 30.4% of those surveyed experienced depression, and 33% experienced anxiety [37]. Also, we found in depression that the majority of the patients, 46.9%, were thought to have extremely low levels of depression, followed by mild depression in 29.4% and moderate depression in 11.2% of them. However, another 8.2% of those who underwent bariatric surgery were thought to have moderately high depression levels, and 4.4% had severe depression during the study. In a study that was conducted in the USA, it was found that the percentage of depression after bariatric surgery was 32% [37].

Women account for the majority of participants, 58%. According to some theories, women in Saudi Arabia and other nations like the US and Canada seek health services at a higher rate than men due to their greater care for their health [38], desire for a beauty standard, and positive traits [39]. 

Also, the results indicate a significant weight loss when comparing the BMI before and after surgery. Additionally, the patient's BMI has improved by 94%, and they have not reverted to their preoperative levels. Another study confirms this: those who underwent bariatric surgery lost more weight than those who underwent non-surgical treatment, proving the surgery's favorable results [40].

We observed improvements in all obesity-related health problems, particularly snoring and sleep apnea, which decreased by 40.9% and 34.1%, respectively. This finding is similar to a study in which >75% of patients had resolution or at least some improvement after BS [40]. In addition, they concluded that BS caused significant weight loss and better sleep [41].

The present study also demonstrates a 48.5% improvement in joint pain, and comparable investigations have shown that this improvement occurred over 12 months and was maintained for three years. Furthermore, bariatric surgery can correct pre-osteoarthritis risk factors and aberrant joints [42]. 

Our study found that 20.7% of participants had worsening esophageal regurgitation. While earlier research has shown that 27.9% of patients had GERD symptoms [43], laparoscopic sleeve gastrectomy (LSG) impacts lower esophageal sphincter (LES) pressure by changing the His angle and severing ligaments, contributing to GERD's high prevalence [44].

Menstrual disorders affected women with PCOS more commonly before BS; 40 patients with ovarian cysts have improved PCOS with BS. Similar to other studies in meta-analysis, 21 studies have demonstrated that PCOS has significantly decreased [45]. In another study following BS, there was a significant increase in the percentage of patients with regular periods in the PCOS group [46]. Therefore, patients with PCOS may benefit particularly from surgical obesity management. 

The complication rate reported by the patient in the current study was 10.4%, comparable to the 8% rate of major complications and the 15% rate of minor complications reported in a multicenter study [47]. The anxiety and depression levels of the patients in the present study did not show any statistically significant changes postoperatively in the short, medium, or long term. Other researchers reported lower rates of symptoms of depression after surgery when compared to before surgery [48], which lasted for two to three years after surgery [49]. Improvement in depressive symptoms following surgery may be attributed to various biological and psychosocial pathways, including weight-related physical conditions, increased daily activity, body image satisfaction, improved cognitive functioning, and better partnership quality and sexual functioning [50-56].

In some studies, the initial postoperative improvement in depressive symptoms [57] did not last, and depression increased after four years. However, using the hospital anxiety and depression scale (HADS), others found that depression scores were comparable to presurgical levels [58,59]. Therefore, the decline in mental health during this time could be caused by factors other than weight regain. Unrealistic expectations or the recurrence of psychiatric disorders could explain the deterioration in mood improvement. Furthermore, changes in eating habits, medical complications following surgery, the fear of regaining weight, dissatisfaction with body appearance and excess skin, or the persistence of problems blamed on obesity may all contribute to the decline.

However, almost all of the patients, 97%, who underwent bariatric surgery in the present study were satisfied with the outcomes. This finding is higher than that found among patients in Taif, 42.6% [60], Najran, 66.9% [61], Saudi Arabia, and Oslo, Norway [62] over a five-year follow-up, which ranged from 90% to 62%.

Limitations

Some potential limitations include: recall bias may result from the complexity and multifactorial nature of depression and anxiety, and many participants with multiple morbidities. Furthermore, we relied on patient memory to obtain a mental health history before the operation, which is susceptible to recall bias. We don't know how applicable the results will be to a larger population because all participants came from a single institution. Furthermore, the PHQ-9 and GAD-7 are depression and anxiety screening instruments, but a definitive diagnosis is still the psychiatrist's opinion.

## Conclusions

Bariatric surgery effectively reduces weight and improves obesity-related health problems, such as snoring, sleep apnea, joint pain, and PCOS-related menstrual disorders. Therefore, patients suffering from these conditions may benefit from surgical obesity management. Anxiety and depression are more common. As a result, we strongly recommend pre- and postoperative psychiatric evaluation for all patients undergoing bariatric surgery to reduce these issues and improve postoperative outcomes.

## Appendices

Appendix 1

The categorical principal component analysis of the patients' economic household income, educational level, and employment as well as their social state showed that these factors had loaded significantly and saliently (well ≥0.512) onto one latent factor that we named as socioeconomic state factor score. This latent factor explained a total of 46% of the shared covariance between the four socioeconomic indicators, which is a substantial explained variance. People who scored higher on this SES score tended to be more (educated, employed, had higher household monthly income, and were married) in general and vice versa. The categorical Cronbach's alpha test of internal consistency was adequate for the four variables: Cronbach's alpha=0.71. This socioeconomic factor score is a standardized z-score that is computed and saved by the statistical data analysis program, and it had a mean score=0 and a standard deviation=1. 

**Table 7:** Categorical principal component analysis yielded loadings for socioeconomic indicators to their latent SES factor. SES: socioeconomic factor.

	Socioeconomic factor (SES) score
Social state=married	0.512
Educational level	0.730
Employment type	0.718
Households' monthly income level	0.717
Variable principal normalization.	
Note: loadings=these numbers are understood just like Pearson's correlation coefficient-r, larger correlation size denotes a stronger association, and it can be bound between −1 and +1.	
